# Development and Evaluation of Copper Based Transparent Heat Reflectors Obtained by Magnetron Sputtering

**DOI:** 10.3390/nano12193544

**Published:** 2022-10-10

**Authors:** Iulian Pana, Anca C. Parau, Mihaela Dinu, Adrian E. Kiss, Lidia R. Constantin, Catalin Vitelaru

**Affiliations:** National Institute of Research and Development for Optoelectronics—INOE 2000, 409 Atomistilor St., 077125 Magurele, Romania

**Keywords:** transparent heat reflectors, magnetron sputtering, optical properties

## Abstract

Within the next few years climate change is likely to become a major concern for mankind. In addition, the current electronic components shortage crisis has led to an urgent need for alternative solutions in the main industry sectors (the raw materials, manufacturing, and construction industries). The current trends of research are focused on developing smart materials with functional properties, using abundant raw materials. The energy saving efforts are sustained in the glazing industries by several approaches based on dielectric-metal-dielectric multilayer structures. The use of silver to achieve a high reflectivity in near-infrared spectral range has been proposed and is already adopted as a commercially available solution. This work is focused on developing a transparent heat reflector (THR) with prefigured optical properties, using copper as a reflective layer, a material that is more abundant and cheaper than silver. The conductive copper layers obtained by the High Power Impulse Magnetron Sputtering (HiPIMS) method were interposed between two silicon nitride layers deposited by the Radio-Frequency Magnetron Sputtering (RFMS) technique. The structural, optical, and elemental composition of monolayers was investigated, qualifying each individual material for use in the multilayer structure. The time stability of films deposited on microscope glass substrates was also investigated, as an important criterion for the selection of monolayers. The obtained results revealed that the SiN_x_/Cu/SiN_x_ with the Cu layer deposited by using a negative substrate bias of −100 V showed the most stable behavior over time. Optical modeling was performed to design a THR multilayer structure, which was successfully obtained experimentally. A maximum optical transparency as high as 75% in the visible range and a reflectivity of ~ 85% in near infrared spectral interval was confirmed for the experimentally obtained multilayer structures.

## 1. Introduction

The increase of thermal efficiency of buildings has been a major topic of interest in the past decades, in the context of increased awareness of energy consumption and its negative impact on the environment [1]. A practical solution to do this is to control the radiation flux through windows [2], as windows are a known source of heat transfer to the outside. The main properties required for such applications are: a good transparency in the visible part of the spectrum, high reflectivity in the infrared domain, thermal and mechanical stability in time, visual aspect that can respond to architectural needs. The types of coatings used for these applications are generally called low-emissivity coatings and they are multilayers that contain a succession of dielectric (D) and metallic materials (M). The minimum structure of such a coating contains at least one of each type of material, with the most common structure being D/M/D [3].

The metallic layer plays a central role in reflecting infrared radiation, being thin enough to ensure good transparency of the assembly in the visible range. The materials used for this purpose are metals with high reflectivity in the infrared domain, such as Au, Cu, Al, Ag [3], in thin layers that are close to the coalescence limit. The most commonly used metal for this purpose is silver [4,5,6], due to its good reflectivity and stability over time. Nevertheless, there is a high interest in finding alternative materials with similar properties, which are cheaper and can be found easily in nature. One such example is copper [7,8,9], which has the potential advantage of forming a continuous layer at thicknesses that are smaller than those of silver [10], or even improving the silver layer properties and continuity at equivalent thickness when used as a seed layer [11]. Copper has been used in combination with various dielectric materials, such as ZrO_2_ [12], TiO_2_ [13], ZnO [14], etc. Moreover, systematic modeling was performed to investigate the potential use of a large variety of protective layers, such as SiO_2_, MgO, AZO, ITO, ZnO, NiO, MoO_2_, TeO_2_, Nb_2_O_5_, ZnS, TiO_2_ [15]. The inferior stability of Cu to oxidation can represent a serious drawback for these applications, being known that the thickness of growing oxide can reach a few nanometers in a matter of days [16], due to the native oxide layer, which is not self-protective against Cu oxidation [17]. One way to improve oxidation resistance over time is by increasing the bias voltage during deposition [17]. Additionally, using HiPIMS (High Power Impulse Magnetron Sputtering) [18,19,20] for the deposition of metallic thin films leads to higher available ion fluxes to the substrate [21]. The superior stability of HiPIMS obtained copper vs. DC sputtered, using −100V bias voltage, was already demonstrated in Ref. [22].

The dielectric layers have a double role, being protective for the metallic layer and also improving the optical transparency, especially in the visible range. Generally, oxide layers are used [6,15], combinations of oxides and nitrides [23,24], or nitrides such as SiN_x_ [24,25].

There are various versions of the multilayer structure used, the elementary unit being formed by one metal layer and one dielectric transparent layer. The complexity can be increased by introducing two or more metallic layers [24] or by introducing interlayers between the metallic and the dielectric layers [25]. The overall properties of the multilayer can be adjusted by the variation of individual layer thickness [13,14,26], or by applying thermal treatments after the deposition [13,26].

In this study we used a copper layer as a heat reflective element in a multilayer D/M/D structure containing SiN_x_ as the dielectric layer. The stability of Cu was ensured both by the deposition process, namely HiPIMS with substrate biasing, and the use of a nitride as a protective dielectric in an oxygen free process.

## 2. Materials and Methods

**Figure 1:**
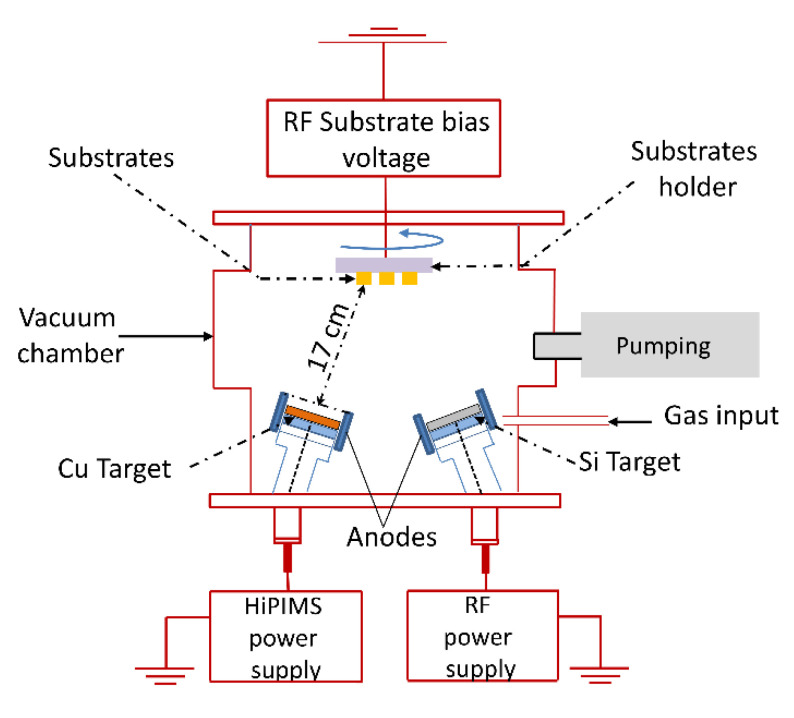
Experimental setup for mono- and multilayer deposition.

The Cu and SiN_x_ thin layers were obtained by using an AJA-ATC-ORION magnetron sputtering deposition unit (AJA International Inc., Scituate, MA, USA) (Figure 1). The deposition chamber was equipped with 5 individual magnetron cathodes that were positioned in a confocal geometry and could be used either simultaneously or independently. The diameter of each cathode was 50.8 mm and only two cathodes (Si and Cu) were used for obtaining the thin films described in this work. Prior to each deposition run, the substrates were ultrasonically cleaned with isopropyl alcohol for 15 min. After this step, the substrates were introduced in the deposition chamber and were sputter cleaned in an Ar^+^ plasma for 30 min (f = 13.56 MHz, P = 50 W, U_bias_ = −305 V, p = 5 mTorr, without any intentional heating of the substrate holder). Three types of substrates were used: microscope glass slides (10 mm × 15 mm, 1 mm thickness) and silicon <111> (10 mm × 15 mm, 0.5 mm thickness) for the deposition of monolayers and samples used for the characterization of individual materials and quartz (10 mm × 15 mm, 0.7 mm thickness) for the deposition of designed multilayers. The vacuum chamber was evacuated down to 1 × 10^−7^ mBar before each deposition process. The SiN_x_ thin film was obtained by sputtering the Si target (Kurt Lesker, Jefferson Hills, PA, USA, 99.99% purity) under RF biasing, at 90 W power, 8.5 sccm of Ar flow and 1.5 sccm of N_2_ flow, respectively, at a constant pressure of 5 mTorr. During the SiN_x_ deposition the substrate holder was not biased. The Cu deposition was performed under HiPIMS sputtering regime, at a peak voltage of 860 V, a pulse current of 15 A, a pulse duration of 50 μs, and a frequency of 25 Hz. A constant pre-ionization current of 3 mA was used to ensure the conditions for pulse initiation and stable functioning. The substrate bias voltage was used as a control parameter for adjusting the properties of the Cu layer interposed between 2 dielectric layers of SiN_x_. The bias voltage values investigated in this work were 0, −50, and −100 V, respectively.

The thickness, density, and roughness of multilayers were investigated by X-ray reflectivity (XRR) technique, by using a SmartLab diffractometer (RIGAKU, Tokyo, Japan), equipped with a rotating Cu anode (9 kW) and 5-axis vertical goniometer. The diffractometer was operated using high-resolution optics on the incident beam (Ge (220) × 2 monochromator) in order to obtain the Cu-Kα_1_ radiation (λ = 1.540597 Å). The measurements were performed in a 2theta range of 0.3–1.5°, with a speed of 0.16°/min and a resolution of 0.004°.

The optical properties of mono- and multilayers were investigated by using a Jasco V-670 spectrophotometer (Jasco, Tokyo, Japan) in ultraviolet-visible-near infrared spectral range. The spectrophotometer was equipped with an ARSN-733 auxiliary unit (Jasco, Tokyo, Japan) in order to record the experimental transmission and reflectivity curves. The optical layout of ARSN-733 unit implies the use of one concave mirror that reflects the beam after passing within sample and an integrating sphere with a diameter of 60 mm. According to the specifications of the integrating sphere manufacturer, the relative errors associated with absolute reflectivity measurements are ±1.5% at incidence angles of 5°. The scan speed was 400 nm/min and the investigated spectral interval ranged between 250 and 2000 nm.

The infrared measurements were performed on samples deposited on silicon substrates by using the FT-IR Jasco 6300 spectrometer (Jasco, Tokyo, Japan). In this case, a transmittance configuration was used in order to investigate IR spectral response in the 4000–400 cm^−1^ range at a spectral resolution of 4 cm^−1^ and 150 average scans. The sample and detector chamber were continuously purged by using a constant flow of nitrogen gas during the measurements, along with a pre-purging of the interferometer compartment. Prior to any sample measurement, the silicon substrate was used within the background measurement in order to obtain the final IR sample spectra.

## 3. Results

The results are divided into two main parts, the first referring to the characterization of the materials or monolayers that enter the composition of the multilayer structure and the second referring to the design, deposition, and characterization of the multilayer structure.

### 3.1. Material Deposition and Characterization

#### 3.1.1. Overall Time Stability of Transparent Heat Reflector Structures

**Figure 2:**
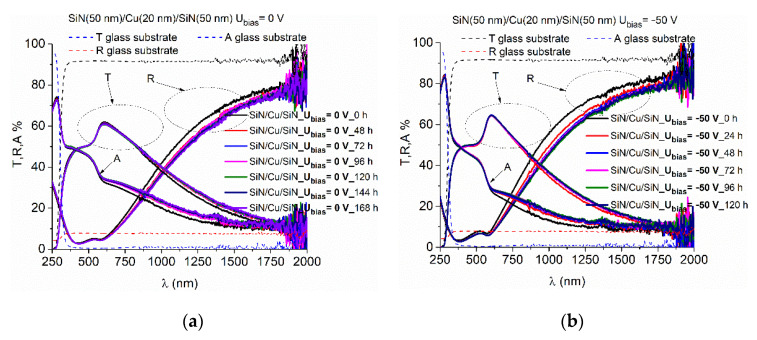

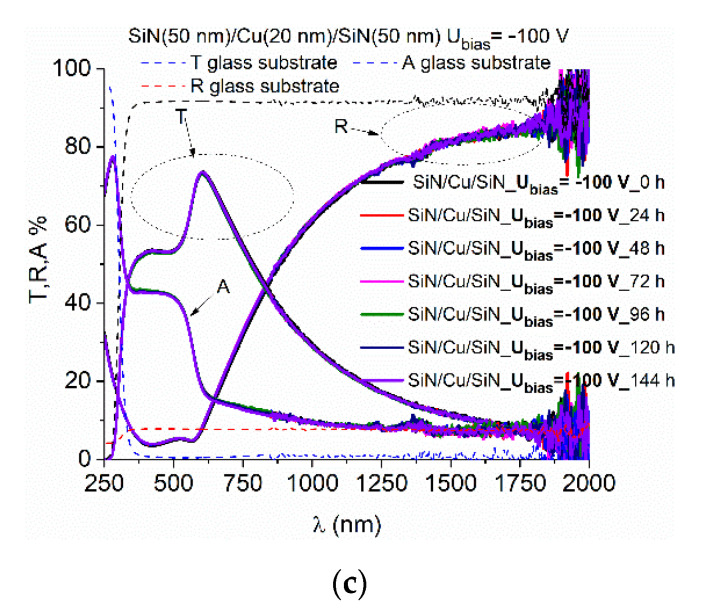
Variation of experimental transmission, reflectance and absorption spectra of multilayer thin films SiN_x_(50 nm)/Cu(20 nm)/SiN_x_(50 nm) obtained at different substrate bias voltages: (**a**) 0 V, (**b**) −50 V, (**c**) −100 V after exposure to atmospheric pressure conditions.

The use of Cu as a reflecting layer was previously assessed [22] and the stability of Cu deposited directly on glass substrate was investigated. Besides the improvement of the optical characteristics of the multilayer, the role of the dielectric layer is also to protect the Cu layer. Therefore, in this work we investigated the stability of a multilayer structure containing SiN_x_/Cu/SiN_x_ layers. For this purpose, samples were prepared by using the same sputtering conditions for the dielectric layer, whereas the Cu layer was obtained at different bias voltages (0 V, −50 V, and −100 V, respectively). The thickness of individual layers was 50 nm for both SiN_x_ layers and 20 nm for the Cu layer. The time evolution of reflectivity, transmittance, and absorption for three such structures are represented in Figure 2. The profiles obtained right after removing the samples from the vacuum chamber can be identified with “0 h”, whereas the other profiles are marked with the corresponding number of hours between the first measurement and the current one. The profiles were registered at regulated intervals of approximately 24 h from the moment they were brought out from the vacuum chamber. The performance of the multilayers varies as follows:

(i) For the multilayer containing the Cu layer obtained at 0 V bias voltage, one can see an important degradation of the optical properties as an increase of absorption and a decrease of reflectivity in the near infrared domain.
(ii) For the multilayer containing the Cu layer obtained at −50 V bias voltage, the most significant degradation can be observed. Initially this multilayer has a smaller absorption as compared with the previous case but a more pronounced degradation in the first 24 h after deposition;
(iii) The multilayer containing the Cu layer obtained at −100 V bias voltage is the most stable among all, with no significant changes being observed in the first 144 h from removing the samples from the vacuum chamber.

**Figure 3:**
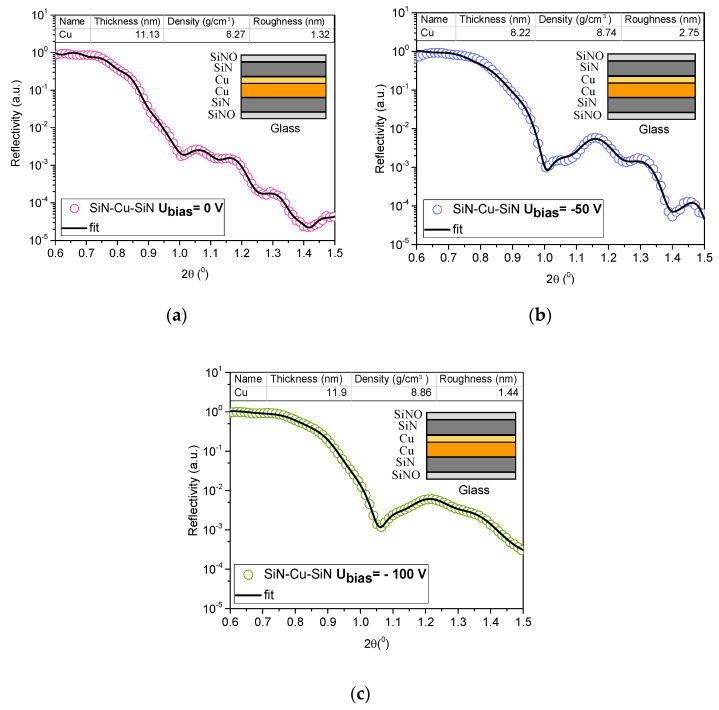
XRR spectra of SiN_x_/Cu/SiN_x_ multilayers at the following substrate bias voltages (**a**) 0 V, (**b**) −50 V and (**c**) −100 V.

The XRR spectra obtained for the SiN_x_/Cu/SiN_x_ structures obtained at different bias voltages during Cu deposition are presented in Figure 3. The thickness, density and roughness values resulted from fitting the reflectivity spectra are also presented, together with the model structure used for modeling. When a multilayer structure is analyzed by XRR technique, due to the fact that each layer has a different electronic density, the reflected X-rays will interfere, resulting in an interference induced oscillation pattern’’. When no sharp interfaces are present, the number of interface fringes gradually reduces or they become almost invisible, giving an indication for a possible deterioration of interfaces. One can see in Figure 3 that the number of oscillations decreases with increasing bias voltage, almost vanishing completely for the sample obtained at −100 V. The correlation between the quality of the layer interface and the XRR pattern was investigated by An et al. [27], showing the effect of a higher bias voltage in the case of TiN/SiN_x_ coatings deposited by magnetron sputtering. It is known that by applying a higher bias voltage to the substrate, a larger number of ions can be accelerated toward the substrate, thus assisting the forming layer with a higher energy [28,29]. Nevertheless, a value of the bias voltage that is too high can lead to excessive interdiffusion between layers, due to the high intensity ionic bombardment. The density of Cu layer increases with increasing bias voltage, starting from 8.27 g/cm^3^ (0 V) up to 8.86 g/cm^3^ (−100 V), the density obtained being very close to the refence density of Cu (8.96 g/cm^3^). This density increase can be explained by the increased energy of impinging ions during deposition, and corresponding increase of adsorbed atoms mobility on the surface [30]. Similar results for the density dependency of Cu layers on the bias voltage were previously obtained by Choi et al. [31]. The difference in the trend of evolution of roughness of the Cu layer obtained at bias voltage −50 V and −100 V can be explained by film growth and the competition between deposition and re-sputtering mechanisms. The increase of bias voltage over a certain limit, where re-sputtering becomes important, leads to an enhanced particle movement with a beneficial effect on density and a lower surface roughness. The effect of the applied bias on the microstructure was previously reported by Yubao et al. [32].

According to the structure obtained after the fitting, there is an interlayer between the main Cu layer and the SiN_x_ layer, with a thickness between 2 and 5 nm. This layer is also affected by the bias voltage variation, having a smaller density as compared with the Cu layer, varying between 4.59 g/cm^3^ and 6.96 g/cm^3^.

The analysis of the time stability of the structure confirmed that the Cu layer deposited at −100 V bias voltage under HiPIMS conditions offers the highest stability. Therefore, in the following, these deposition conditions were used for obtaining the multilayer structure.

#### 3.1.2. Optical Characteristics of Monolayer Materials

**Figure 4:**
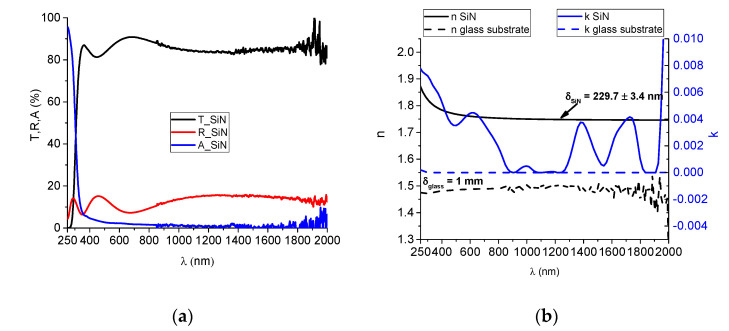
(**a**) Experimental spectrophotometric curves and (**b**) dispersion curves for the refractive index and extinction coefficient.

SiN_x_ layers were deposited on microscope glass, 1 mm thick, according to the process condition described in the experimental setup section, using a deposition time of 240 min. The spectrophotometric curves are represented in Figure 4a, indicating a good transparency of the material and reflectivity values smaller than 15%. The accurate estimation of refractive index and extinction coefficient was performed by using complex algorithms of the OptiChar module of the OptiLayer optical modeling software [33]. This implies the evaluation initially of the optical properties of the substrate as a function of its thickness. Then, the transmission and reflectivity data of the SiN_x_ layer are introduced as input parameters in a non-parametric model based on the second order derivative. This model aims to obtain a minimum characteristic of a merit function, with a desired absolute function smaller than 1.41 [34]. The resulting refractive index variation in the 250 to 2000 nm interval is represented in Figure 4. The thickness resulted from optical modeling is 229.7 nm, being also depicted in Figure 4. The refractive index is approximately 1.75, smaller than the refractive index obtained for Si_3_N_4_, which was 1.98 [35]. This indicates that the material is under-stoichiometric. The extinction coefficient is smaller than 0.004 in the visible spectral range, with slightly higher values in the near UV domain, thus qualifying this material as optically non-absorbent.

**Figure 5:**
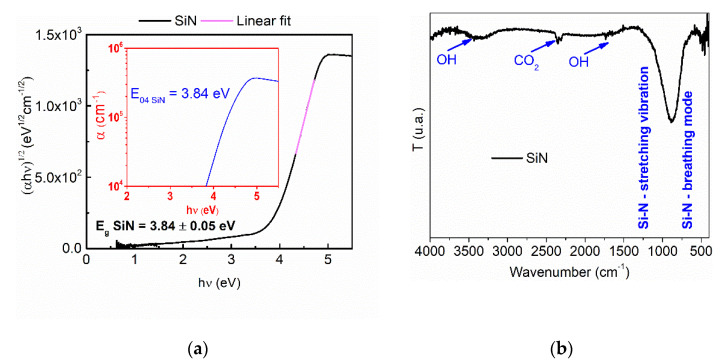
(**a**) Tauc plot for indirect band gap of SiN_x_ monolayer with photon energy dependence of absorption coefficient (E_04_ = f (hν)—inset) and (**b**) FT-IR transmission spectrum of SiN_x_ film.

The optical bandgap was determined by using the Tauc algorithm [36], taking into account the photon absorption in the sample. For this purpose, the spectrophotometric curves and the layers thickness obtained by optical modeling in OptiChar module at previous step were used. It is known that the silicon nitride possesses indirect transitions, and by extrapolating the slope of the linear fitting resulted for (αhν)^1/2^ = 0, the value of the optical bandgap was determined to be 3.84 eV, as illustrated in Figure 5a. This value is smaller as compared with the bulk material SiN (~ 5 eV) and then the reference values that can be found in the literature for the stoichiometric Si_3_N_4_ (between 4.5 and 4.7 eV) [37].

Following the analysis of FT-IR spectra of the sample obtained on Si substrate, few chemical bonds specific to the SiN_x_ layers were obtained. Two absorption bands can be identified and they are marked with blue vertical lines in Figure 5b. These absorption bands observed in the FT-IR spectra are centered on the wavenumbers at 916 cm^−1^ and 443 cm^−1^ and were attributed to asymmetric stretching vibrations characteristic to the Si-N group [38]. There is a good agreement between values of refractive index, optical bandgap and the main stretching vibration band located at 916 cm^−1^ which strengthens the assumption that the SiN_x_ coating is under-stoichiometric.

**Figure 6:**
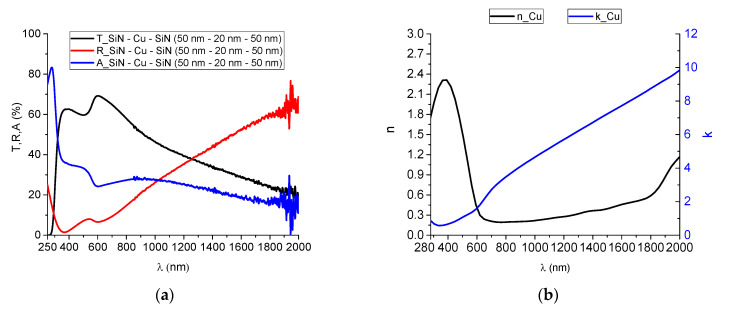
(**a**) Experimental spectrophotometric curves of the multilayer obtained on microscope glass substrate and (**b**) dispersion curves of the refractive index and absorption coefficient of the Cu layer between two SiN_x_ layers.

The optical properties of the metallic layer can be different depending on the substrate on which it grows. Therefore, in order to simulate as close as possible the conditions in the future multilayer structure, a Cu layer of 20 nm was deposited between two SiN_x_ layers, having a thickness of 50 nm each. For the deposition of the Cu layer, the experimental conditions described in the “Materials and Methods” section were used, with a bias voltage of −100 V on the substrate. The spectrophotometric curves of this structure are represented in Figure 6a. The optical properties of the SiN_x_ layers were considered as known parameters and introduced in the OptiRe module of the OptiLayer optical modeling software [39], along with the spectrophotometric curves in Figure 6a. The resulting dispersion curves for the Cu layer are represented in Figure 6b.

### 3.2. Optical Characteristic of the Designed Multilayer Structure

**Figure 7:**
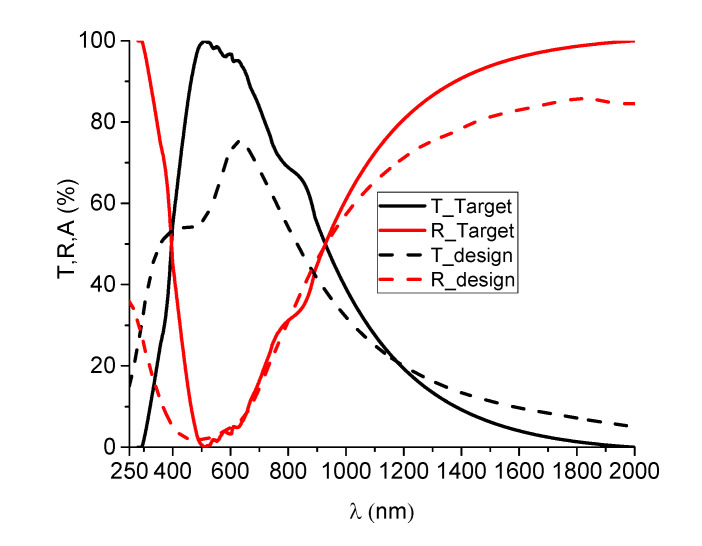
Spectrophotometric curves of the desired target and the optimized THR structure.

The dispersion curves obtained for SiN_x_ (Figure 4b) and Cu (Figure 6b) were used as input parameters in the OptiLayer modeling software. A multilayer structure was designed based on these optical characteristics, through successive optimization steps and taking into account the characteristics needed for a transparent heat reflector. The target used in the modeling process was the solar emission spectrum, the ideal targeted design having the characteristic of letting pass the entire solar spectrum and reflecting infrared radiation. The desired target and the optimized design optical characteristics are represented in Figure 7. The resulting structure consisted of two SiN_x_ layers with thicknesses of 36 and 58 nm, respectively, and a Cu layer of 30 nm thickness.

**Figure 8:**
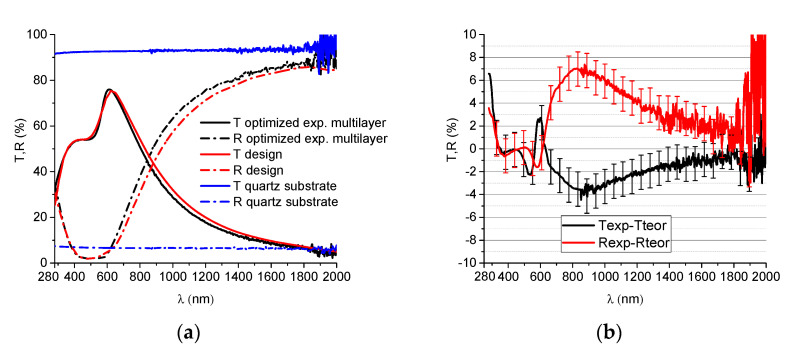
(**a**) Comparison of theoretical and experimental spectrophotometric curves of the optimized THR structure obtained on fused quartz and (**b**) the wavelength dependency of the difference between reflectivity and transmittance of experimental versus theoretical curves.

The designed structure resulted from modeling and was obtained experimentally, by successive deposition of the three layers, without breaking the vacuum. The spectrophotometric curves of the designed and experimentally obtained structure are represented in Figure 8, along with the characteristic curves of quartz substrate. By comparing the two sets of curves, one can see that there is a good agreement between the designed and experimentally obtained properties. The mathematical difference between the designed and experimental curves are represented in Figure 8b, showing a difference as high as 4% for the transmittance and 7% for the reflectivity. There are several reasons for which there is a difference between the experiment and design. Besides the small errors or variations that can occur due to the deposition process, the difference can also come from the initial evaluation of the optical properties of individual layers. By using the spectrophotometric data of SiN_x_ on glass we assumed that the properties of the dielectric layer are the same in the multilayer structure. This is not entirely valid due to the possible intermixing with the Cu layer and to the growth of SiN_x_ on Cu instead of glass, for the second SiN_x_ layer. Nevertheless, this assumption is necessary in order to evaluate the unknown parameters of Cu. When interpreting the differences between those curves, one should also take into account the 1.5% measurement error indicated by the spectrophotometer manufacturer (indicated as error bars in Figure 8b). We can conclude that the differences are small and the designed structure was successfully obtained experimentally, with an error in the order of ~5%.

## 4. Discussion

A combined theoretical and experimental approach was used to design and experimentally obtain a transparent heat reflective structure. The use of copper as an alternative material reflective element in a D/M/D structure was investigated. The use of HiPIMS provided the means to enhance the quality and stability of the metallic thin film, by adjusting the bias voltage and corresponding energy of the ions impinging on the surface. A bias voltage of −100 V was found to provide better stability of the structure, maintaining the optical parameters for a longer time. The design of the structure was performed using the dispersion curves of the monolayers. In order to account for the variations of film growth on different substrates, the Cu layer was deposited between two dielectric layers, with known properties. Therefore, the optical properties of the metallic thin film can be deduced by using all of the other parameters as known inputs: thickness of individual layers and dispersion curves of dielectric material. The optical properties of the designed and the experimentally obtained structure are compared, revealing differences that are on the order of ~5%, taking into account measurement errors. The results indicate that using copper as a reflective element can be a viable solution for transparent heat reflector coatings. One should take into account the stability of the structure, especially the intermixing of the metallic layer at the interfaces or its oxidation. Using a nitride dielectric instead of oxide can improve the stability, by eliminating oxygen as a process gas from the technological process. Further improvements can be achieved by using proper management of ions fluxes to the substrate, providing film densification and improving stability over time. Future developments should include improvements of the optical properties of the materials by further adjusting the deposition parameters. Moreover, more complicated designs can be envisaged, using two metallic layers.

## 5. Conclusions

A SiN_x_/Cu/SiN_x_ multilayer structure intended for transparent heat reflector applications was designed, obtained, and characterized. The results indicated the best performance for the multilayer comprising the Cu layer of 30 nm deposited by HiPIMS using a negative substrate bias of −100 V. The Cu layer is surrounded by two dielectric layers with thicknesses of 36 nm (bottom layer) and 58 nm (top layer) and this structure showed the most stable behavior over time, with negligible changes after preserving the samples several days under normal temperature and humidity atmospheric conditions.

## Data Availability

Not applicable.
